# Association of dairy intake with all-cause, cancer, and cardiovascular disease mortality in Japanese adults: a 25-year population-based cohort

**DOI:** 10.1007/s00394-021-02734-6

**Published:** 2021-11-08

**Authors:** Yukai Lu, Yumi Sugawara, Sanae Matsuyama, Akira Fukao, Ichiro Tsuji

**Affiliations:** 1 Division of Epidemiology, Department of Health Informatics and Public Health, Graduate School of Medicine, Tohoku University School of Public Health, 2-1 Seiryo-machi, Aoba-ku, Sendai, 980-8575 Japan; 2 Miyagi Cancer Society, Sendai, Japan

**Keywords:** Dairy, Milk, Yogurt, Cheese, Cancer, Cardiovascular disease, Mortality

## Abstract

**Purpose:**

The association between dairy intake and mortality remains uncertain, and evidence for the Japanese population is scarce. We aimed to investigate the association between dairy intake and all-cause, cancer, and cardiovascular disease (CVD) mortality in Japanese adults.

**Methods:**

A total of 34,161 participants (16,565 men and 17,596 women) aged 40–64 years without a history of cancer, myocardial infarction, or stroke at baseline were included in the analysis, using data from the Miyagi Cohort Study initiated in 1990. Milk, yogurt, and cheese intake were obtained using a validated food frequency questionnaire. Total dairy intake was calculated as the sum of milk, yogurt, and cheese intake and then categorized by quartile. The outcomes were all-cause, cancer, and CVD mortality. Cox proportional hazards regression models were used to estimate multivariable hazard ratios (HRs) and 95% confidence intervals (CIs) for mortality risks.

**Results:**

During 750,016 person-years of follow-up, the total number of deaths was 6498, including 2552 deaths due to cancer and 1693 deaths due to CVD. There was no association between total dairy intake and all-cause, cancer, and CVD mortality for both men and women. We also examined the associations between subgroup dairy products and mortality. For milk and yogurt intake, our results suggest null associations. However, cheese intake was modestly associated with lower all-cause mortality in women; compared with non-consumers, the multivariable HRs (95%CIs) were 0.89 (0.81–0.98) for 1–2 times/month, 0.88 (0.78–1.00) for 1–2 times/week, and 0.89 (0.74–1.07) for 3 times/week or almost daily (*p* trend = 0.016).

**Conclusion:**

Dairy intake was not associated with mortality in Japanese adults, except for limited evidence showing a modest association between cheese intake and a lower all-cause mortality risk in women.

**Supplementary Information:**

The online version contains supplementary material available at 10.1007/s00394-021-02734-6.

## Introduction

Dairy products contribute various valuable nutrients to the overall diet, including protein, vitamins, and minerals, and consumption of dairy products is recommended in most dietary guidelines worldwide [1]. Previous studies have suggested that associations between dairy intake and multiple health outcomes, including diabetes mellitus [2], cardiovascular diseases (CVD) [3], breast cancer [4, 5], and colorectal cancer [5, 6], are null or weak inverse. Minerals in milk such as calcium, potassium, and magnesium may have played a role in the effect of milk on reducing blood pressure, which then may contribute to lowering the risk of CVDs [7]. However, recent randomized controlled trials have shown dairy-rich diet has no effect on blood pressure compared to dairy-free diet [8, 9]. Also, dairy products have a high content of saturated fat which raises low-density lipoprotein cholesterol level, consequently contributing to higher risk of CVDs [10], but current evidence has shown that neither whole milk nor low-fat milk has been clearly associated with CVDs [11]. Moreover, calcium in milk is related to protecting against the breast cancer and colorectal cancer risks [5].

However, it is possible that high consumption of dairy foods is associated with increased risks of prostate cancer [5, 12]. Greater concentrations of insulin-like growth factor I (IGF-I) has been associated with the elevated prostate cancer risk, and milk consumption may increase IGF-I blood concentrations [13]. Evidence also has showed that total dairy intake is associated with a higher risk of endometrial cancer, particularly among postmenopausal women who are not currently using hormone therapy [14], which may attribute to the sex-hormone content of dairy products such as estrogen [15]. Therefore, whether dairy intake is beneficial or harmful to health is controversial due to the various nutrients in dairy products.

Numerous meta-analyses have investigated the association between dairy intake and mortality, but their results have been controversial [16–24]. Some suggested that higher dairy intake was associated with a lower mortality risk [16, 23], whereas others suggested a null association [17–19, 21, 22, 24]. Larsson et al. argued that it was perhaps inappropriate to pool the results due to their considerable heterogeneity [20]. It should be mentioned that most studies are conducted in Europe or North America where dairy products are traditionally consumed far more than in other regions [16]. Several cohort studies from Japan examining dairy intake and mortality showed inconsistent results [25–27]. One study found that milk drinking was associated with a lower risk of CVD mortality and cancer mortality in men [27], but the other two suggested null associations [25, 26]. Thus, whether different patterns of dairy intake between Western and Asian populations may be associated with mortality differently has been unclear. It also needs to be noted that different kinds of dairy products vary in their nutrient composition, so they may have different effects on health [16]. For example, previous studies suggested that yogurt or cheese rather than milk intake was associated with a lower risk of all-cause mortality [16, 28, 29].

Thus, the aim of the present study was to examine the association of dairy intake with all-cause, cancer, and CVD mortality in Japanese adults, using a large-scale population-based cohort with follow-up over 25 years.

## Materials and methods

### Study participants

**Fig. 1:**
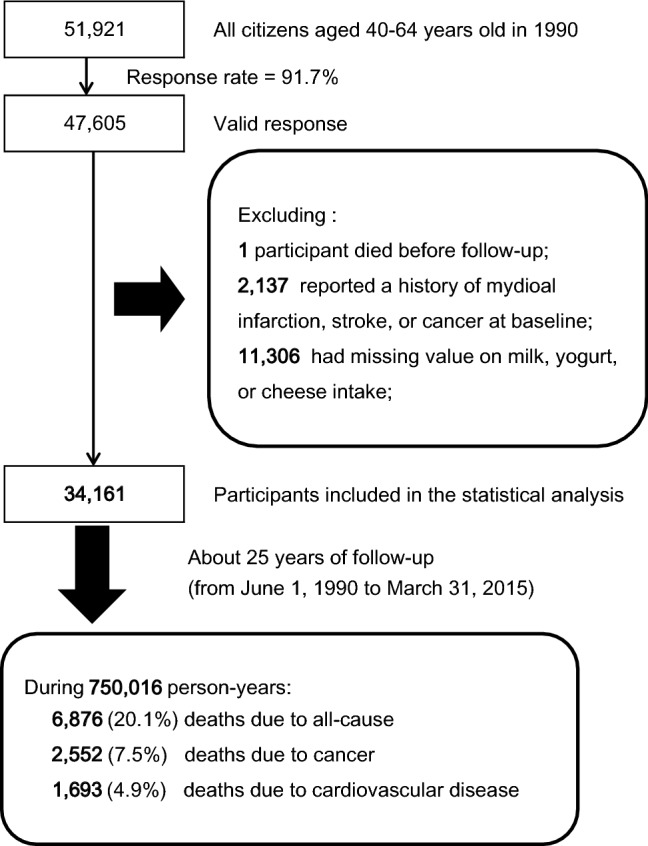
Flowchart of study participants

The data used in the present study came from the Miyagi Cohort, the design of which has been described in detail elsewhere [30, 31]. In brief, between June and August 1990, a self-administered questionnaire on various health conditions was delivered to all residents aged 40–64 years (*n* = 51,921) in 14 municipalities of Miyagi Prefecture, northeastern Japan. Of them, 47,605 were confirmed to be eligible (response rate: 91.7%) (Fig. 1).

For the present analysis, one participant withdrawn from the study before follow-up starting, 2137 participants who had a history of cancer, myocardial infarction, or stroke at baseline, and 11,306 persons who did not answer the questions on milk, yogurt, or cheese intake were excluded. Eventually, 34,161 responses (16,565 men and 17,596 women) were included for the present analysis.

The study protocol was approved by the institutional review board of the Tohoku University School of Medicine (Approval No. 2014–1-838). We considered the return of self-administered questionnaires signed by the participants to imply their consent to participate in the study.

### Dietary assessment and dairy intake (exposure)

Participants were asked about the average intake of dairy products including milk, yogurt, and cheese, as well as other food items, during the previous year, using a validated food frequency questionnaire (FFQ). The FFQ included 39 food items and several beverages. For dairy products intake, participants were required to choose from the following five categories: “almost never”, “1–2 times/month”, “1–2 times/week”, “3–4 times/week”, and “almost daily”.

We also conducted a validation study for the FFQ we used for this study previously [32]. The age- and total energy-adjusted Spearman’s correlation coefficients between 3-day diet records and the FFQ were 0.72 for milk, 0.56 for yogurt, and 0.36 for cheese in men, and 0.65 for milk, 0.60 for yogurt, and 0.36 for cheese in women.

The volume of each food intake was calculated by converting the intake frequency from the FFQ into a daily intake volume (g/day). Daily intake was calculated by multiplying the average number of daily servings (times/day) by an assigned portion size (g/time) from the FFQ based on the median values observed in the validation study. Total dairy intake was calculated as the sum of daily intake of milk, yogurt, and cheese and was then sex-specifically categorized by quartile, with Q1 the lowest quartile and Q4 the highest one. For estimation of energy and other nutrient intakes from the food intake volume based on the FFQ, a food composition table that corresponded to the items listed in the questionnaire was used. This food composition table was developed using the Standard Tables of Food Composition published by the Science and Technology Agency of Japan [33].

### Follow-up

The primary outcomes were all-cause, cancer, and CVD mortality, and the secondary outcomes were coronary heart disease (CHD) and stroke morality as well as lung cancer, gastric cancer, and colorectal cancer mortality. To follow-up the participants for mortality and migration, we established a Follow-up Committee [30, 34, 35], consisting of the Miyagi Cancer Society, the Community Health Divisions of all 14 municipalities, the Department of Health and Welfare of Miyagi Prefectural Government, and the Division of Epidemiology, Tohoku University Graduate School of Medicine. The Committee periodically reviewed the Residential Registration Record of each municipality. With this review, we identified participants who had either died or emigrated during the follow-up period. We discontinued follow-up with those who had emigrated from the study area, because the Committee could not review the Residential Registration Record from outside the study area.

For identified decedents, we further investigated cause of death by reviewing the death certificates of the participants at Public Health Centres in the study area. The cause of death was defined according to the International Classification of Diseases (ICD) 9th revision (ICD-9) between June 1, 1990 and December 31, 1998 and the 10th revision (ICD-10) between January 1, 1999 and March 31, 2015. Death due to CVD was coded by ICD-9:390-459 or ICD-10:I00-I99 (CHD: ICD-9:410-414 or ICD-10:I20-I25; stroke:ICD-9:430-438 or ICD-10:I60-I69), and death due to cancer was coded by ICD-9:140–239 or ICD-10:C00-D09 (lung cancer:ICD-9:162 or ICD-10:C34; gastric cancer:ICD-9:151 or ICD-10:C16; colorectal cancer:ICD-9:153-154 or ICD-10:C18-C20).

Participants were followed up from June 1, 1990 to March 31, 2015. The number of person-years of follow-up for each participant was counted from the beginning of follow-up until the date of death, the date of emigration from the study districts, or the end of follow-up, whichever occurred first. During the follow-up period, 2997 participants (8.8%) were lost to follow-up.

### Statistical analysis

Cox proportional hazards model was used to calculate the sex-specific hazard ratios (HRs) and 95% confidence intervals (95% CIs) for mortality according to the quartile of total dairy intake, with participants in the lowest quartile (Q1) as the reference. Dummy variables were created for each group of exposure and categorical covariates. Missing values of each covariate were classified into an extra group. Time of follow-up was used as the time scale. Multivariable models were adjusted as follows: Model 1 was adjusted for age (continuous); Model 2 was further adjusted for education level (junior high school or lower, high school, college or higher, or missing), BMI (< 18.5 kg/m^2^, 18.5–24.9 kg/m^2^, ≥ 25.0 kg/m^2^, or missing), smoking status (never, former, < 20 cigarettes/day, ≥ 20 cigarettes/day, or missing), alcohol drinking status (never, former, current, or missing), and history of disease [hypertension and diabetes mellitus (yes or no for each)]; Model 3 was further adjusted for energy intake (in tertiles, or missing), vegetable and fruit intake (in tertiles, or missing), and fish intake (in tertiles, or missing). In addition, a test for trend was also conducted by coding the exposure variable using the median value of each category in the models.

A sensitivity analysis was also conducted by excluding deaths in the initial three years of follow-up, considering the possible reverse causality where health condition at baseline may affect dairy intake. Then, several stratified analyses according to age (< 50 vs. ≥ 50 y), BMI (< 25.0 vs. ≥ 25.0 kg/m^2^), and smoking status (current vs. non-current) were also conducted, because these covariates which have a great impact on health outcomes may differ the association between dairy intake and mortality. A test for interaction was also performed by adding an additional cross-product term of exposure variable and stratified covariate to the models. Because 11,306 persons with missing data on milk, yogurt, or cheese were excluded from the present analysis, which may affect the results, multiple imputations for missing data on dairy products were also applied. Five datasets with missing values being imputed according to age, sex, and other covariates, and Cox models were then created and applied to calculate the pooled HRs and 95% CIs for mortality using the five imputed datasets [36].

Milk, yogurt, and cheese intake frequency were categorized into four groups based on FFQ responses. To obtain sufficient participants in each group, we combined “almost never” and “1–2 times/month” for milk, and “3–4 times/week” and “almost daily” for yogurt and cheese. Butter was not included because it is distinct from other dairy foods in nutritional components and its correlation between 3-day diet records and the FFQ (0.20 for men and 0.11 for women) was much lower than other dairy products. All analyses were repeated using each dairy product intake frequency as the exposure variable, with the least frequent group as the reference.

All analyses were performed using SAS version 9.4 (SAS Inc., Cary, NC, USA). All statistical tests described were two-sided, and differences at *p* < 0.05 were considered statistically significant.

## Results

### Baseline characteristics

During 750,016 person-years of follow-up, the total number of deaths was 6876 (4354 men and 2522 women), including 2552 deaths due to cancer (1713 men and 839 women) and 1693 deaths due to CVD (1048 men and 645 women). Table 1 shows the baseline characteristics by total dairy intake. The mean (standard deviation) total dairy intake was 125.9 (93.9) g/day for men and 148.1 (94.9) g/day for women, which was similar to that of general Japanese population (mean 130.1 g/day) [37] but was less than half of the amount in western countries measured in previous studies [38–42]. In both men and women, people with higher dairy intake were more likely to have a high education level, to be never smokers, to have high energy intake, or to have high vegetable and fruit intake.

**Table 1:** Characteristics at baseline according to total dairy intake (*n* = 34,161)

	Quartile of total dairy intake^β^			
	Q1	Q2	Q3	Q4
Men (*n* = 16,565)				
No. of participants	3979	4195	3366	5025
Dairy intake (g/day)^α^	6.6 (7.5)	71.5 (27.7)	180.0 (41.8)	229.7 (26.7)
Range of quartiles (g/day)	0–40.8	44.9–109.8	110.8–210.0	211.0–325.0
Age (years)^α^	50.1 (7.4)	49.7 (7.3)	51.2 (7.5)	50.7 (7.4)
College or higher (%)^β^	12.6	14.8	16.1	19.3
BMI (kg/m^2^)^α^	23.5 (2.8)	23.7 (2.8)	23.7 (2.8)	23.5 (2.6)
Never smokers (%)	14.8	18.0	20.1	23.3
Never alcohol drinkers (%)	14.3	13.6	15.9	16.6
Time spent walking (> 1 h/day) (%)	44.5	42.2	44.5	42.5
Energy intake (kJ/day)^α^	7126.9 (2545.4)	7496.3 (2443.0)	7742.4 (2386.4)	8050.9 (2376.2)
Fish intake (g/day)^α^	56.7 (35.3)	58.7 (33.5)	63.3 (34.7)	63.0 (34.2)
Vegetable and fruit intake (g/day)^α^	146.8 (101.1)	165.8 (100.9)	188.4 (110.0)	216.9 (112.9)
History of hypertension (%)	17.1	17.5	17.2	16.8
History of diabetes (%)	3.5	4.2	6.5	5.9
Women (*n* = 17,596)				
No. of participants	4396	4135	4514	4551
Dairy intake (g/day)^α^	17.5 (19.0)	103.8 (32.1)	212.9 (2.6)	250.1 (27.0)
Range of quartiles (g/day)	0–49.4	49.9–205.0	210.0–224.5	229.3–310.0
Age (years)^α^	51.1 (7.4)	49.8 (7.1)	52.2 (7.2)	50.6 (7.3)
College or higher (%)^β^	9.6	13.8	14.4	19.1
BMI (kg/m^2^)^α^	23.8 (3.3)	23.6 (3.1)	23.7 (3.0)	23.5 (3.0)
Never smokers (%)	86.3	89.8	90.7	92.2
Never alcohol drinkers (%)	69.6	69.3	71.6	68.9
Time spent walking (> 1 h/day) (%)	44.0	44.2	45.1	42.2
Energy intake (kJ/day)^α^	5108.0 (1390.5)	5528.2 (1321.6)	5819.0 (1353.1)	6050.7 (1292.3)
Fish intake (g/day)^α^	49.6 (29.5)	53.8 (28.6)	56.3 (29.2)	59.1 (28.7)
Vegetable and fruit intake (g/day)^α^	213.7 (116.9)	246.4 (111.4)	252.5 (112.8)	291.7 (112.9)
History of hypertension (%)	20.8	17.1	19.1	17.8
History of diabetes (%)	2.2	1.8	3.8	3.1

^α^Mean (standard deviation) for all such values

^β^Aged ≥ 19 y when participants had completed their education

### Dairy intake and mortality

The sex-specific associations between total dairy intake and all-cause, cancer, and CVD mortality are presented in Table 2. However, there were no associations between total dairy intake and all-cause, cancer, and CVD mortality in both men and women. Tables 3, 4 and 5 show the associations between milk, yogurt, and cheese intakes and mortality, respectively. Similarly, our results suggest null associations between milk or yogurt intake and all-cause, cancer, and CVD mortality in both men and women (Tables 3, 4). However, for cheese intake (Table 5), a modest association with a lower risk of all-cause mortality was observed in women; compared with non-consumers, the multivariable HRs (95%CI) were 0.89 (0.81–0.98) for 1–2 times/month, 0.88 (0.78–1.00) for 1–2 times/week, and 0.89 (0.74–1.07) for 3 times/week or almost daily (*p* trend = 0.016). We also examined the association between dairy intake and secondary outcomes including CHD and stroke mortality, as well as lung cancer, gastric cancer, and colorectal cancer mortality, but no association was found (e-Tables 1&2).

**Table 2:** Association between total dairy intake and mortality (*n* = 34,161)^α^

	Quartile of total dairy intake^β^				*P *trend^γ^
	Q1	Q2	Q3	Q4	
Men					
Person-years	84,007	89,576	71,387	1,08,493	
All-cause mortality					
No. of death	1102	1023	956	1273	
Model 1^δ^	1.00 (ref.)	0.91 (0.84–0.99)	0.95 (0.87–1.03)	0.84 (0.78–0.92)	0.003
Model 2^ε^	1.00 (ref.)	0.94 (0.86–1.02)	0.98 (0.90–1.07)	0.91 (0.84–0.99)	0.174
Model 3^ζ^	1.00 (ref.)	0.94 (0.87–1.03)	0.98 (0.90–1.07)	0.93 (0.85–1.01)	0.328
Cancer mortality					
No. of death	437	421	363	492	
Model 1^δ^	1.00 (ref.)	0.95 (0.83–1.08)	0.91 (0.79–1.04)	0.83 (0.73–0.94)	0.006
Model 2^ε^	1.00 (ref.)	0.98 (0.85–1.12)	0.95 (0.82–1.09)	0.90 (0.79–1.02)	0.121
Model 3^ζ^	1.00 (ref.)	0.99 (0.86–1.13)	0.96 (0.83–1.10)	0.92 (0.81–1.05)	0.237
CVD mortality					
No. of death	268	232	236	312	
Model 1^δ^	1.00 (ref.)	0.86 (0.72–1.02)	0.96 (0.80–1.14)	0.85 (0.72–1.00)	0.339
Model 2^ε^	1.00 (ref.)	0.88 (0.74–1.05)	0.99 (0.83–1.18)	0.93 (0.79–1.10)	0.978
Model 3^ζ^	1.00 (ref.)	0.88 (0.74–1.05)	0.99 (0.83–1.18)	0.94 (0.79–1.11)	0.972
Women					
Person-years	98,927	93,883	1,01,276	1,02,466	
All-cause mortality					
No. of death	659	518	731	614	
Model 1^δ^	1.00 (ref.)	0.93 (0.83–1.05)	1.00 (0.90–1.11)	0.94 (0.84–1.05)	0.644
Model 2^ε^	1.00 (ref.)	0.97 (0.87–1.09)	1.04 (0.94–1.16)	0.98 (0.88–1.10)	0.722
Model 3^ζ^	1.00 (ref.)	0.98 (0.87–1.10)	1.05 (0.94–1.17)	1.00 (0.89–1.12)	0.574
Cancer mortality					
No. of death	228	161	235	215	
Model 1^δ^	1.00 (ref.)	0.82 (0.67–1.01)	0.94 (0.79–1.13)	0.94 (0.78–1.14)	0.723
Model 2^ε^	1.00 (ref.)	0.85 (0.69–1.04)	0.97 (0.80–1.16)	0.98 (0.81–1.18)	0.970
Model 3^ζ^	1.00 (ref.)	0.85 (0.69–1.04)	0.98 (0.81–1.17)	0.99 (0.82–1.21)	0.899
CVD mortality					
No. of death	170	133	192	150	
Model 1^δ^	1.00 (ref.)	0.96 (0.77–1.21)	1.00 (0.81–1.23)	0.90 (0.72–1.12)	0.586
Model 2^ε^	1.00 (ref.)	1.01 (0.81–1.27)	1.05 (0.86–1.30)	0.94 (0.75–1.17)	0.917
Model 3^ζ^	1.00 (ref.)	1.03 (0.82–1.30)	1.06 (0.86–1.31)	0.95 (0.76–1.20)	0.976

^α^Hazard ratios (HRs) and 95% confidence intervals (95% CIs) were calculated by Cox proportional hazards models

^β^Ranges for the quartiles of total dairy intake were 0–40.8 g/day, 44.9–109.8 g/day, 110.8–210.0 g/day, and 211.0–325.0 g/day in men and 0–49.4 g/day, 49.9–205.0 g/day, 210.0–224.5 g/day, and 229.3–310.0 g/day in women

^γ^*P trend* was calculated using the median value of each category of total dairy intake

^δ^Model 1 was adjusted for age (continuous)

^ε^Model 2 was adjusted for Model 1 plus education level (junior high school or lower, high school, college or higher, or missing), BMI (< 18.5 kg/m^2^, 18.5–24.9 kg/m^2^, ≥ 25.0 kg/m^2^, or missing), smoking status (never, former, < 20 cigarettes/day, ≥ 20 cigarettes/day, or missing), alcohol drinking status (current, never, former, or missing), history of hypertension (yes, or no), and history of diabetes (yes, or no)

^ζ^Model 3 was adjusted for Model 2 plus energy intake (sex-specific tertiles or missing), fish intake (sex-specific tertiles or missing),and vegetable and fruit intake (sex-specific tertiles or missing)

**Table 3:** Association between milk intake and mortality (*n* = 34,161)^α^

	Milk intake frequency				*P *trend^β^
	Almost never/1–2 times/mo	1–2 times/week	3–4 times/week	Almost daily	
Men					
Person-years	86,587	55,599	57,796	1,53,481	
All-cause mortality					
No. of death	1131	635	632	1956	
Model 1^γ^	1.00 (ref.)	0.94 (0.85–1.04)	0.88 (0.80–0.97)	0.90 (0.84–0.97)	0.004
Model 2^δ^	1.00 (ref.)	0.96 (0.87–1.06)	0.92 (0.83–1.01)	0.95 (0.88–1.02)	0.184
Model 3^ε^	1.00 (ref.)	0.97 (0.88–1.06)	0.93 (0.84–1.02)	0.96 (0.89–1.04)	0.339
Cancer mortality					
No. of death	451	255	274	733	
Model 1^γ^	1.00 (ref.)	0.95 (0.81–1.11)	0.95 (0.82–1.11)	0.85 (0.75–0.95)	0.005
Model 2^δ^	1.00 (ref.)	0.97 (0.83–1.13)	1.00 (0.86–1.16)	0.91 (0.80–1.02)	0.107
Model 3^ε^	1.00 (ref.)	0.98 (0.84–1.14)	1.01 (0.87–1.18)	0.93 (0.82–1.04)	0.214
CVD mortality					
No. of death	271	139	150	488	
Model 1^γ^	1.00 (ref.)	0.86 (0.71–1.06)	0.87 (0.72–1.07)	0.93 (0.80–1.08)	0.504
Model 2^δ^	1.00 (ref.)	0.89 (0.72–1.09)	0.92 (0.75–1.12)	1.00 (0.86–1.16)	0.794
Model 3^ε^	1.00 (*ref.*)	0.89 (0.72–1.09)	0.92 (0.76–1.13)	1.00 (0.86–1.17)	0.787
Women					
Person-years	77,460	51,494	63,856	2,03,743	
All-cause mortality					
No. of death	527	295	355	1345	
Model 1^γ^	1.00 (ref.)	0.99 (0.86–1.14)	0.92 (0.80–1.05)	0.97 (0.88–1.08)	0.572
Model 2^δ^	1.00 (ref.)	1.00 (0.87–1.16)	0.96 (0.84–1.10)	1.01 (0.92–1.12)	0.785
Model 3^ε^	1.00 (ref.)	1.00 (0.87–1.16)	0.97 (0.84–1.11)	1.02 (0.92–1.14)	0.633
Cancer mortality					
No. of death	183	96	110	450	
Model 1^γ^	1.00 (ref.)	0.90 (0.70–1.15)	0.80 (0.63–1.02)	0.94 (0.79–1.11)	0.574
Model 2^δ^	1.00 (ref.)	0.91 (0.71–1.17)	0.83 (0.65–1.05)	0.96 (0.81–1.15)	0.822
Model 3^ε^	1.00 (ref.)	0.92 (0.72–1.18)	0.84 (0.66–1.06)	0.98 (0.82–1.17)	0.950
CVD mortality					
No. of death	132	78	93	342	
Model 1^γ^	1.00 (ref.)	1.09 (0.82–1.44)	0.99 (0.76–1.29)	0.99 (0.81–1.21)	0.732
Model 2^δ^	1.00 (ref.)	1.10 (0.83–1.46)	1.06 (0.81–1.38)	1.04 (0.84–1.27)	0.888
Model 3^ε^	1.00 (ref.)	1.11 (0.84–1.47)	1.08 (0.82–1.41)	1.05 (0.85–1.30)	0.771

^α^Hazard ratios (HRs) and 95% confidence intervals (95% CIs) were calculated by Cox proportional hazards models

^β^*P* trend was calculated by treating exposure as a continuous variable

^γ^Model 1 was adjusted for age (continuous)

^δ^Model 2 was adjusted for Model 1 plus education level (junior high school or lower, high school, college or higher, or missing), BMI (< 18.5 kg/m^2^, 18.5–24.9 kg/m^2^, ≥ 25.0 kg/m^2^, or missing), smoking status (never, former, < 20 cigarettes/day, ≥ 20 cigarettes/day, or missing), alcohol drinking status (current, never, former, or missing), history of hypertension (yes, or no), and history of diabetes (yes, or no)

^ε^Model 3 was adjusted for Model 2 plus energy intake (sex-specific tertiles or missing), protein intake (sex-specific tertiles or missing), fish intake (sex-specific tertiles or missing),and vegetable and fruit intake (sex-specific tertiles or missing)

**Table 4:** Association between yogurt intake and mortality (*n* = 34,161)^α^

	Yogurt intake frequency				*P *trend^β^
	Almost never	1–2 times/mo	1–2 times/wk	3 times/week/ Almost daily	
Men					
Person-years	2,02,265	82,965	43,393	24,840	
All-cause mortality					
No. of death	2680	883	458	333	
Model 1^γ^	1.00 (ref.)	0.85 (0.79–0.92)	0.83 (0.75–0.92)	0.95 (0.85–1.07)	0.001
Model 2^δ^	1.00 (ref.)	0.90 (0.83–0.97)	0.88 (0.80–0.97)	1.02 (0.91–1.14)	0.111
Model 3^ε^	1.00 (ref.)	0.91 (0.84–0.98)	0.90 (0.81–0.99)	1.04 (0.92–1.17)	0.253
Cancer mortality					
No. of death	1048	348	191	126	
Model 1^γ^	1.00 (ref.)	0.86 (0.76–0.97)	0.89 (0.76–1.04)	0.92 (0.77–1.11)	0.064
Model 2^δ^	1.00 (ref.)	0.90 (0.79–1.01)	0.95 (0.81–1.11)	1.00 (0.83–1.20)	0.473
Model 3^ε^	1.00 (ref.)	0.91 (0.80–1.03)	0.97 (0.83–1.14)	1.03 (0.85–1.24)	0.791
CVD mortality					
No. of death	647	213	112	76	
Model 1^γ^	1.00 (ref.)	0.86 (0.73–1.00)	0.85 (0.69–1.04)	0.90 (0.71–1.14)	0.064
Model 2^δ^	1.00 (ref.)	0.92 (0.79–1.08)	0.91 (0.74–1.11)	0.99 (0.78–1.25)	0.448
Model 3^ε^	1.00 (ref.)	0.93 (0.80–1.09)	0.91 (0.75–1.12)	0.99 (0.78–1.26)	0.488
Women					
Person-years	1,30,658	1,06,634	94,770	64,491	
All-cause mortality					
No. of death	987	612	538	385	
Model 1^γ^	1.00 (ref.)	0.89 (0.80–0.98)	0.91 (0.82–1.01)	0.88 (0.78–0.99)	0.027
Model 2^δ^	1.00 (ref.)	0.91 (0.82–1.01)	0.94 (0.84–1.04)	0.91 (0.81–1.03)	0.109
Model 3^ε^	1.00 (ref.)	0.91 (0.83–1.01)	0.94 (0.85–1.05)	0.92 (0.81–1.03)	0.146
Cancer mortality					
No. of death	307	209	177	146	
Model 1^γ^	1.00 (ref.)	0.95 (0.80–1.13)	0.94 (0.78–1.13)	1.06 (0.87–1.29)	0.825
Model 2^δ^	1.00 (ref.)	0.97 80.82–1.16)	0.96 (0.80–1.16)	1.08 (0.88–1.32)	0.641
Model 3^ε^	1.00 (ref.)	0.98 (0.82–1.17)	0.97 (0.81–1.18)	1.10 (0.89–1.34)	0.541
CVD mortality					
No. of death	262	157	131	95	
Model 1^γ^	1.00 (ref.)	0.89 (0.73–1.08)	0.88 (0.71–1.08)	0.84 (0.67–1.07)	0.110
Model 2^δ^	1.00 (ref.)	0.92 (0.75–1.12)	0.89 (0.72–1.10)	0.86 (0.68–1.09)	0.160
Model 3^ε^	1.00 (ref.)	0.93 (0.76–1.14)	0.91 (0.73–1.12)	0.87 (0.69–1.11)	0.221

^α^Hazard ratios (HRs) and 95% confidence intervals (95% CIs) were calculated by Cox proportional hazards models

^β^*P* trend was calculated by treating exposure as a continuous variable

^γ^Model 1 was adjusted for age (continuous)

^δ^Model 2 was adjusted for Model 1 plus education level (junior high school or lower, high school, college or higher, or missing), BMI (< 18.5 kg/m^2^, 18.5–24.9 kg/m^2^, ≥ 25.0 kg/m^2^, or missing), smoking status (never, former, < 20 cigarettes/day, ≥ 20 cigarettes/day, or missing), alcohol drinking status (current, never, former, or missing), history of hypertension (yes, or no), and history of diabetes (yes, or no)

^ε^Model 3 was adjusted for Model 2 plus energy intake (sex-specific tertiles or missing), fish intake (sex-specific tertiles or missing),and vegetable and fruit intake (sex-specific tertiles or missing)

**Table 5:** Association between cheese intake and mortality (*n* = 34,161)^α^

	Cheese intake frequency				*P *trend^β^
	Almost never	1–2 times/mo	1–2 times/wk	3 times/wk/ Almost daily	
Men					
Person-years	1,67,125	1,25,487	45,575	15,276	
All-cause mortality					
No. of death	2276	1345	523	210	
Model 1^γ^	1.00 (ref.)	0.87 (0.81–0.93)	0.90 (0.82–1.00)	0.98 (0.85–1.13)	0.018
Model 2^δ^	1.00 (ref.)	0.89 (0.83–0.95)	0.94 (0.85–1.03)	1.03 (0.89–1.18)	0.158
Model 3^ε^	1.00 (ref.)	0.89 (0.83–0.96)	0.96 (0.87–1.05)	1.05 (0.91–1.22)	0.356
Cancer mortality					
No. of death	838	566	231	78	
Model 1^γ^	1.00 (ref.)	0.99 (0.89–1.11)	1.09 (0.94–1.26)	0.99 (0.79–1.25)	0.548
Model 2^δ^	1.00 (ref.)	1.00 (0.90–1.12)	1.11 (0.96–1.29)	1.03 (0.81–1.30)	0.322
Model 3^ε^	1.00 (ref.)	1.01 (0.91–1.13)	1.15 (0.99–1.33)	1.08 (0.85–1.36)	0.140
CVD mortality					
No. of death	573	309	115	51	
Model 1^γ^	1.00 (ref.)	0.80 (0.70–0.92)	0.79 (0.65–0.97)	0.95 (0.71–1.26)	0.017
Model 2^δ^	1.00 (ref.)	0.82 (0.72–0.95)	0.86 (0.70–1.05)	1.00 (0.75–1.34)	0.114
Model 3^ε^	1.00 (ref.)	0.83 (0.72–0.95)	0.86 (0.70–1.06)	1.01 (0.75–1.34)	0.136
Women					
Person-years	1,89,818	1,24,799	60,612	21,323	
All-cause mortality					
No. of death	1385	685	324	128	
Model 1^γ^	1.00 (ref.)	0.86 (0.79–0.94)	0.84 (0.75–0.95)	0.85 (0.71–1.02)	0.001
Model 2^δ^	1.00 (ref.)	0.89 (0.81–0.98)	0.88 (0.78–0.99)	0.89 (0.74–1.07)	0.013
Model 3^ε^	1.00 (ref.)	0.89 (0.81–0.98)	0.88 (0.78–1.00)	0.89 (0.74–1.07)	0.016
Cancer mortality					
No. of death	437	236	122	44	
Model 1^γ^	1.00 (ref.)	0.92 (0.79–1.08)	0.98 (0.80–1.20)	0.92 (0.67–1.25)	0.536
Model 2^δ^	1.00 (ref.)	0.95 (0.81–1.11)	1.02 (0.83–1.25)	0.95 (0.70–1.30)	0.819
Model 3^ε^	1.00 (ref.)	0.96 (0.81–1.12)	1.03 (0.84–1.27)	0.95 (0.70–1.31)	0.923
CVD mortality					
No. of death	354	172	83	36	
Model 1^γ^	1.00 (ref.)	0.88 (0.73–1.05)	0.88 (0.69–1.12)	0.94 (0.67–1.33)	0.254
Model 2^δ^	1.00 (ref.)	0.90 (0.75–1.09)	0.93 (0.73–1.18)	0.99 (0.70–1.40)	0.546
Model 3^ε^	1.00 (ref.)	0.91 (0.76–1.10)	0.95 (0.74–1.21)	0.99 (0.70–1.41)	0.634

^α^Hazard ratios (HRs) and 95% confidence intervals (95% CIs) were calculated by Cox proportional hazards models

^β^*P* trend was calculated by treating exposure as a continuous variable

^γ^Model 1 was adjusted for age (continuous)

^δ^Model 2 was adjusted for Model 1 plus education level (junior high school or lower, high school, college or higher, or missing), BMI (< 18.5 kg/m^2^, 18.5–24.9 kg/m^2^, ≥ 25.0 kg/m^2^, or missing), smoking status (never, former, < 20 cigarettes/day, ≥ 20 cigarettes/day, or missing), alcohol drinking status (current, never, former, or missing), history of hypertension (yes, or no), and history of diabetes (yes, or no)

^ε^Model 3 was adjusted for Model 2 plus energy intake (sex-specific tertiles or missing), fish intake (sex-specific tertiles or missing),and vegetable and fruit intake (sex-specific tertiles or missing)

### Sensitivity analyses

We conducted a sensitivity analysis by excluding deaths in the initial three years of follow-up, but the results were not essentially changed for total dairy intake and subgroup dairy products (e-Tables 3 and 4). We also conducted stratified analyses by age, BMI, and smoking status (e-Tables 5–8), and the results did not differ by those stratified variables. Moreover, we applied multiple imputation for missing values for dairy products and re-analyzed the imputed data, but the pattern of observed results remained for both total dairy intake and subgroup dairy products (e-Tables 9 and 10).

## Discussion

### Main findings

The present study examined the associations of both total dairy intake and subgroup dairy products with all-cause, cancer, and CVD mortality using a large-scale cohort study of the Japanese population with a follow-up period up to 25 years. The results suggested that dairy intake was not associated with mortality, except for limited evidence showing a modest association between cheese intake and a lower risk of all-cause mortality in Japanese women.

### Comparisons with previous studies

There were no associations between total dairy intake and all-cause, cancer, and CVD mortality, which were in line with previous meta-analyses [16, 18, 24]. One suggested that total dairy products intake (per 200 g/day) was not associated with all-cancer mortality risk (RR: 0.99, 95% CI 0.96–1.03), but there was considerable heterogeneity (*I*^2^ = 62.2%, *p* = 0.005) [18]. The other study found that the highest group of total dairy intake was not associated with cancer mortality (RR: 0.99, 95% CI 0.92–1.07) compared to the lowest group [24]. Most cohort studies included in the meta-analyses were from Western populations, and no meta-analyses reported results stratified by study regions. We identified several cohort studies from Asia [26, 43–45]. One study from Japan showed that consumption of milk and dairy products (per 100 g/day) was inversely associated with CVD mortality risk in women (HR: 0.86, 95% CI 0.74–0.99), but not in men [26]. Studies from Taiwan (0 vs. > 7 times/week) and Iran (per serving/day; 230 g for milk and yogurt and 28 g for cheese) both suggested that total dairy intake was inversely associated with all-cause and CVD mortality, but not cancer mortality [43, 44], and the Iranian study also found that the association was more apparent for low-fat dairy intake [44]. Another study from Singapore suggested a marginally significant inverse association between total dairy intake and CVD mortality, especially stroke mortality, but only in men and those without a prior history of CVD [45].

Milk intake was not associated with all-cause, cancer, and CVD mortality in the present study. Numerous meta-analyses generally reported null associations [16, 18, 19, 21, 22, 24], but studies conducted in Asian populations showed controversial results [27, 44, 46, 47]. For all-cause mortality, four meta-analyses found no association with milk intake [18, 19, 21, 22], although three of them had considerable heterogeneity (*I*^2^: 72.3–97.4%) [18, 19, 22]. Two cohort studies from Iran and China suggested no association between milk and all-cause mortality [44, 46], whereas one Japanese study found that milk intake was associated with a lower risk only in men aged 65 years or older, but not in women [27]. An Iranian study suggested that consuming whole milk daily or more was associated with a higher risk of all-cause mortality compared to non-consumers, but they only included a small number of study participants [47]. For cancer mortality, one meta-analysis reported a null association with milk intake [24], which was also suggested in an Iranian study [44]. The Japanese study found that people drinking milk had a lower risk of cancer mortality only among men aged 65 years or older, but the association was not linear [27]. In contrast, the study from China reported that high milk consumption (> 3 servings/week; 1 serving = 250 ml) was associated with a higher cancer mortality risk [46], but the association between milk intake and cancer is rather complex considering the different effects on various site-specific cancers [48]. For CVD mortality, one meta-analysis suggested that there was no significant association with milk intake, but high heterogeneity was observed [21]. Several studies from Asian regions also did not find an increased risk of CVD mortality in people with higher milk intake [44, 46, 47]. The inconsistency in the milk–mortality association among previous studies may be attributable to different milk intake assessments (e.g., times/week, servings/day, or g/day) and amounts of milk intake, the inclusion of different confounders in the models, various follow-up periods, or variations in age and the number of study participants.

For yogurt intake, we did not find any associations with mortality in both men and women. The present study is the first to examine the association between yogurt intake and all-cause and CVD mortality in the Japanese population. Previous meta-analyses did not find yogurt to be associated with the risk of mortality [18, 24, 49], but one reported large heterogeneity (*I*^2^ = 65.8%, *p* = 0.054) across studies included [18]. One meta-analysis presented subgroup results according to study regions (4 studies from Europe and 2 studies from Asia), and no association was found for both regions [49]. One of the two Asian studies was from Iran and reported a modest inverse association between yogurt intake (per serving/day; 1 serving: 230 g) and all-cause and CVD mortality, but not cancer mortality [44], whereas the other from Japan only investigated the association with cancer mortality, and no association was found (daily vs. not daily) [25]. Several recent cohort studies did not show clear associations between yogurt intake and mortality risks in US and European adults [16, 28, 50, 51]. However, one multinational cohort study including both Western and Eastern populations suggested that yogurt consumption was inversely associated with all-cause mortality, although it was uncertain whether the observation was consistent across different regions [52].

The only modest association in the present study was observed between cheese intake and all-cause mortality in Japanese women. No other studies have examined the association between cheese intake and all-cause, cancer or CVD mortality in the Japanese population. Less evidence was available compared to other dairy products. Two meta-analyses both indicated that cheese intake had no association with all-cause mortality; one included 11 (RR for per 10 g/day: 0.99, 95% CI 0.96–1.01) and the other included 9 (RR for per 50 g/day: 1.03, 95% CI 0.99–1.07) cohort studies [18, 53], but one observed significant heterogeneity (*I*^2^:93.3%, *p* < 0.001) [18]. Several cohort studies were published recently, and three agreed that cheese intake was inversely associated with all-cause mortality [16, 44, 54], whereas two other studies suggested a null association [28, 50]. One meta-analysis examining the association between cheese intake and cancer mortality found no association, although the number of studies included was limited [24]. As for CVD mortality, previous studies agreed that the association between cheese intake and CVD mortality was null or modestly inverse [28, 39, 40, 50, 54, 55].

### Possible mechanisms

The underlying mechanism between cheese intake and all-cause mortality remains unknown, but it is suggested that cheese is rich in numerous nutrients such as whey protein or vitamin K2. Whey protein may have beneficial effects on reducing cardiovascular risk factors, such as improving glucose levels, insulin response and lipid profile as well as lowering blood pressure and controlling body weight [56–58]. Vitamin K2, which is exclusively synthesized by bacteria and predominantly found in fermented foods like cheese, has been shown to plays an important role in preventing metabolic syndrome [59] and CVDs [60–62], although the relation between Vitamin K2 and mortality has shown inconsistency [60, 63, 64]. Moreover, prior studies found that probiotic bacteria in fermented dairy products were reported to have positive effects on immunity, inflammation, diarrhea prevention, and cardiovascular risk factors in clinical trials [3]. Fermented dairy product intake was also inversely associated with all-cause mortality [18, 38], but one study found a marginally inverse association with cheese, but not yogurt, by examining different types of fermented food in relation to all-cause mortality [18]. More speculatively, the lack of association with yogurt may be attributed to the fact that many yogurt products on the market have a certain amount of sugar added [1]. In addition, other than the isolated effect from individual nutrient, current evidence has indicated that the matrix effect of hard cheese may have benefits in reducing the amount of fat absorbed and lowering the blood cholesterol response [65, 66], ultimately leading to an improvement in cardiovascular health [67]. Nevertheless, the observed association between cheese intake and all-cause mortality in the present study could simply be a chance finding due to tests of multiple outcomes. It also needs caution that the correlation between dietary records and the FFQ of cheese was lower than that of milk or yogurt in the present study, which suggested a poorer validity of cheese intake possibly owing to the low intake amount in the study population. Thus, more studies are warranted to confirm the present observation.

### Limitation and strengths

Our study has some strengths, including a large number of study participants, a long period of follow-up, and a high follow-up rate. Meanwhile, some limitations should also be mentioned. First, dairy intake was obtained from a self-reported FFQ, so the misclassification of exposure would be possible. Second, dairy intake was assessed only once, at baseline, but it may change over time during follow-up. Third, details of types of dairy products (e.g., high-fat or low-fat dairy products) were not obtained, and different types of dairy products may affect health diversely. Finally, although a considerable number of covariates were adjusted in our models, residual and unmeasured confounding may still have affected the results.

## Conclusion

The results of the present study suggested that dairy intake was not associated with all-cause, cancer, and CVD mortality in Japanese adults, except for limited evidence showing a modest association between cheese intake and a lower risk of all-cause mortality in women.

## Supplementary Information

Below is the link to the electronic supplementary material.

Supplementary file1 (PDF 370 kb)

## Data Availability

Data described in the manuscript, code book, and analytic code will not be made publicly available because private information of participants were included but are available from the corresponding author on reasonable request.

## Abbreviations

ANOVA: Analysis of variance
CHD: Coronary heart disease
CI: Confidence interval
CVD: Cardiovascular disease
FFQ: Food frequency questionnaire
HR: Hazard ratio
MI: Myocardial infarction
