# Supplementary material for: Association of dairy intake with all-cause, cancer, and cardiovascular disease mortality in Japanese adults: a 25-year population-based cohort
Source: Eur J Nutr. 2021 Nov 8;61(3):1285–97. doi: 10.1007/s00394-021-02734-6 (PMC8921048; doi:10.1007/s00394-021-02734-6)
Supplement: Supplementary file 1 — Supplementary file1 (PDF 370 kb) [file 394_2021_2734_MOESM1_ESM.pdf]

e-Table 1. Association between total dairy intake and sub outcomes among Men (n=16,565)[α]

	Quartile of total dairy intake [β]				*P*-trend [γ]
	Q1	Q2	Q3	Q4	
**CVD mortality**					
CHD [δ]	1.00 (*ref.*)	0.79 (0.55-1.14)	1.10 (0.78-1.55)	0.85 (0.60-1.20)	0.791
Stroke [δ]	1.00 (*ref.*)	0.89 (0.68-1.17)	0.84 (0.63-1.11)	0.97 (0.75-1.25)	0.619
**Cancer mortality**					
Lung cancer [δ]	1.00 (*ref.*)	0.94 (0.71-1.24)	0.90 (0.68-1.21)	0.79 (0.59-1.05)	0.148
Gastric cancer [δ]	1.00 (*ref.*)	1.09 (0.79-1.51)	1.07 (0.77-1.50)	0.90 (0.65-1.25)	0.583
Colorectal cancer [δ]	1.00 (*ref.*)	0.74 (0.47-1.17)	0.79 (0.50-1.26)	1.07 (0.71-1.60)	0.751
	Milk intake frequency				
	Almost never/ 1-2 times/mo	1-2 times/wk	3-4 times/wk	Almost daily	
**CVD mortality**					
CHD [δ]	1.00 (*ref.*)	0.82 (0.54-1.26)	0.73 (0.47-1.14)	1.07 (0.78-1.43)	0.588
Stroke [δ]	1.00 (*ref.*)	0.95 (0.70-1.28)	0.93 (0.69-1.27)	0.93 (0.74-1.18)	0.585
**Cancer mortality**					
Lung cancer [δ]	1.00 (*ref.*)	0.94 (0.69-1.29)	0.93 (0.68-1.28)	0.84 (0.65-1.08)	0.168
Gastric cancer [δ]	1.00 (*ref.*)	1.14 (0.79-1.63)	1.04 (0.72-1.51)	0.94 (0.70-1.26)	0.531
Colorectal cancer [δ]	1.00 (*ref.*)	0.74 (0.44-1.24)	0.61 (0.35-1.07)	1.04 (0.72-1.51)	0.663
	Yogurt intake frequency				
	Almost never	1-2 times/mo	1-2 times/wk	3 times/wk/ Almost daily	
**CVD mortality**					
CHD [δ]	1.00 (*ref.*)	0.77 (0.56-1.07)	0.61 (0.38-0.98)	0.86 (0.51-1.42)	0.062
Stroke [δ]	1.00 (*ref.*)	0.93 (0.72-1.18)	1.06 (0.79-1.44)	1.24 (0.88-1.75)	0.333
**Cancer mortality**					
Lung cancer [δ]	1.00 (*ref.*)	0.81 (0.62-1.06)	0.98 (0.70-1.37)	1.02 (0.68-1.53)	0.753
Gastric cancer [δ]	1.00 (*ref.*)	0.77 (0.57-1.04)	0.77 (0.52-1.15)	1.13 (0.74-1.71)	0.557
Colorectal cancer [δ]	1.00 (*ref.*)	0.71 (0.47-1.06)	0.88 (0.54-1.45)	0.50 (0.23-1.07)	0.058
	Cheese intake frequency				
	Almost never	1-2 times/mo	1-2 times/wk	3 times/wk/ Almost daily	
**CVD mortality**					
CHD [δ]	1.00 (*ref.*)	0.77 (0.57-1.03)	0.96 (0.64-1.43)	1.30 (0.77-2.20)	0.961
Stroke [δ]	1.00 (*ref.*)	0.97 (0.79-1.19)	0.83 (0.60-1.14)	0.83 (0.51-1.37)	0.233
**Cancer mortality**					
Lung cancer [δ]	1.00 (*ref.*)	0.89 (0.70-1.13)	1.23 (0.91-1.67)	1.45 (0.94-2.26)	0.126
Gastric cancer [δ]	1.00 (*ref.*)	0.89 (0.68-1.15)	0.85 (0.58-1.24)	1.17 (0.71-1.95)	0.734
Colorectal cancer [δ]	1.00 (*ref.*)	0.91 (0.64-1.30)	1.49 (0.97-2.28)	1.01 (0.46-2.21)	0.308

[α]Hazard ratios (HRs) and 95% confidence intervals (95%CIs) were calculated by Cox proportional hazards models.

[β]Ranges for the quartiles of total dairy intake were 0-40.8 g/d, 44.9-109.8 g/d, 110.8-210.0 g/d, and 211.0-325.0 g/d in men.

[γ]*P*-trend was calculated using the median value of each category of total dairy intake and calculated by treating exposure as a continuous variable for subgroup dairy products frequency.

[δ]Adjusted for age (continuous),education level (junior high school or lower, high school, college or higher, or missing), BMI (<18.5kg/m^2, 18.5-24.9 kg/m^2, ≥25.0kg/m^2, or missing), smoking status (never, former, <20 cigarettes/d, ≥20 cigarettes/d, or missing), alcohol drinking status (current, never, former, or missing), history of hypertension (yes, or no), history of diabetes (yes, or no), energy intake (sex-specific tertiles or missing), fish intake (sex-specific tertiles or missing), and vegetable and fruit intake (sex-specific tertiles or missing).

e-Table 2. Association between total dairy intake and sub outcomes among Women (n=17,596)[α]

	Quartile of total dairy intake [β]				*P*-trend [γ]
	Q1	Q2	Q3	Q4	
**CVD mortality**					
CHD [δ]	1.00 (ref.)	1.37 (0.87-2.17)	0.95 (0.59-1.53)	0.74 (0.43-1.27)	0.303
Stroke [δ]	1.00 (ref.)	0.95 (0.66-1.35)	1.14 (0.83-1.56)	1.05 (0.75-1.47)	0.460
**Cancer mortality**					
Lung cancer [δ]	1.00 (ref.)	0.80 (0.47-1.37)	0.82 (0.50-1.34)	0.71 (0.42-1.20)	0.252
Gastric cancer [δ]	1.00 (ref.)	0.81 (0.44-1.52)	0.94 (0.53-1.66)	1.37 (0.79-2.37)	0.426
Colorectal cancer [δ]	1.00 (ref.)	0.61 (0.34-1.08)	0.74 (0.45-1.23)	0.98 (0.60-1.61)	0.696
	Milk intake frequency				
	Almost never/ 1-2 times/mo	1-2 times/wk	3-4 times/wk	Almost daily	
**CVD mortality**					
CHD [δ]	1.00 (*ref.*)	1.26 (0.70-2.25)	1.57 (0.92-2.67)	0.91 (0.57-1.45)	0.568
Stroke [δ]	1.00 (*ref.*)	1.07 (0.69-1.66)	1.10 (0.73-1.65)	1.15 (0.83-1.58)	0.272
**Cancer mortality**					
Lung cancer [δ]	1.00 (*ref.*)	1.33 (0.72-2.47)	0.78 (0.40-1.51)	0.85 (0.52-1.39)	0.291
Gastric cancer [δ]	1.00 (*ref.*)	0.78 (0.35-1.72)	0.89 (0.44-1.72)	1.13 (0.66-1.91)	0.482
Colorectal cancer [δ]	1.00 (*ref.*)	0.49 (0.23-1.03)	0.57 (0.30-1.09)	0.77 (0.49-1.19)	0.444
	Yogurt intake frequency				
	Almost never	1-2 times/mo	1-2 times/wk	3 times/wk/ Almost daily	
**CVD mortality**					
CHD [δ]	1.00 (ref.)	1.23 (0.81-1.85)	0.72 (0.43-1.20)	0.71 (0.39-1.27)	0.125
Stroke [δ]	1.00 (ref.)	0.92 (0.68-1.24)	0.98 (0.72-1.34)	1.03 (0.73-1.46)	0.882
**Cancer mortality**					
Lung cancer [δ]	1.00 (*ref.*)	1.10 (0.69-1.75)	0.97 (0.58-1.60)	0.92 (0.52-1.61)	0.737
Gastric cancer [δ]	1.00 (ref.)	0.78 (0.45-1.35)	1.05 (0.61-1.78)	1.20 (0.68-2.12)	0.495
Colorectal cancer [δ]	1.00 (*ref.*)	1.29 (0.79-2.11)	1.63 (0.99-2.66)	1.32 (0.75-2.32)	0.149
	Cheese intake frequency				
	Almost never	1-2 times/mo	1-2 times/wk	3 times/wk/ Almost daily	
**CVD mortality**					
CHD [δ]	1.00 (ref.)	0.95 (0.63-1.42)	0.79 (0.44-1.41)	0.71 (0.28-1.78)	0.323
Stroke [δ]	1.00 (ref.)	0.87 (0.65-1.15)	1.05 (0.74-1.49)	0.96 (0.57-1.61)	0.899
**Cancer mortality**					
Lung cancer [δ]	1.00 (*ref.*)	0.98 (0.64-1.51)	1.16 (0.69-1.96)	0.64 (0.25-1.62)	0.766
Gastric cancer [δ]	1.00 (ref.)	1.32 (0.83-2.09)	1.63 (0.94-2.83)	0.44 (0.11-1.85)	0.538
Colorectal cancer [δ]	1.00 (*ref.*)	1.04 (0.68-1.59)	0.78 (0.42-1.44)	0.88 (0.37-2.06)	0.543

[α]Hazard ratios (HRs) and 95% confidence intervals (95%CIs) were calculated by Cox proportional hazards models.

[β]Ranges for the quartiles of total dairy intake were 0-49.4 g/d, 49.9-205.0 g/d, 210.0-224.5 g/d, and 229.3-310.0 g/d in women.

[γ]*P*-trend was calculated using the median value of each category of total dairy intake and calculated by treating exposure as a continuous variable for subgroup dairy products freqency.

[δ]Adjusted for age (continuous),education level (junior high school or lower, high school, college or higher, or missing), BMI (<18.5kg/m^2, 18.5-24.9 kg/m^2, ≥25.0kg/m^2, or missing), smoking status (never, former, <20 cigarettes/d, ≥20 cigarettes/d, or missing), alcohol drinking status (current, never, former, or missing), history of hypertension (yes, or no), history of diabetes (yes, or no), energy intake (sex-specific tertiles or missing), fish intake (sex-specific tertiles or missing), and vegetable and fruit intake (sex-specific tertiles or missing).

e-Table 3. Association between total dairy intake and mortality after excluding death ascertained in the initial three years among Men (n=16,410)[α]

	Quartile of total dairy intake				*P*-trend [β]
	Q1	Q2	Q3	Q4	
All-cause mortality [γ]	1.00 (*ref.*)	0.94 (0.86-1.03)	1.00 (0.91-1.09)	0.94 (0.86-1.02)	0.654
Cancer mortality [γ]	1.00 (*ref.*)	0.98 (0.85-1.12)	0.97 (0.84-1.12)	0.92 (0.81-1.06)	0.332
CVD mortality [γ]	1.00 (*ref.*)	0.89 (0.74-1.06)	1.01 (0.84-1.21)	0.96 (0.81-1.14)	0.724
	Milk intake frequency				
	Almost never/ 1-2 times/mo	1-2 times/wk	3-4 times/wk	Almost daily	
All-cause mortality [γ]	1.00 (*ref.*)	0.96 (0.87-1.06)	0.93 (0.84-1.03)	0.98 (0.91-1.06)	0.634
Cancer mortality [γ]	1.00 (*ref.*)	0.96 (0.82-1.12)	1.02 (0.88-1.19)	0.93 (0.82-1.05)	0.292
CVD mortality [γ]	1.00 (*ref.*)	0.90 (0.73-1.12)	0.93 (0.76-1.15)	1.03 (0.88-1.20)	0.548
	Yogurt intake frequency				
	Almost never	1-2 times/mo	1-2 times/wk	3 times/wk/ Almost daily	
All-cause mortality [γ]	1.00 (*ref.*)	0.91 (0.84-0.98)	0.90 (0.81-1.00)	1.05 (0.93-1.18)	0.325
Cancer mortality [γ]	1.00 (*ref.*)	0.91 (0.80-1.03)	0.96 (0.82-1.13)	1.05 (0.86-1.27)	0.807
CVD mortality [γ]	1.00 (*ref.*)	0.95 (0.81-1.11)	0.93 (0.76-1.14)	0.96 (0.75-1.23)	0.469
	Cheese intake frequency				
	Almost never	1-2 times/mo	1-2 times/wk	3 times/wk/ Almost daily	
All-cause mortality [γ]	1.00 (*ref.*)	0.90 (0.84-0.97)	0.96 (0.87-1.06)	1.08 (0.93-1.25)	0.603
Cancer mortality [γ]	1.00 (*ref.*)	1.01 (0.91-1.13)	1.13 (0.98-1.32)	1.10 (0.87-1.40)	0.139
CVD mortality [γ]	1.00 (*ref.*)	0.85 (0.74-0.98)	0.88 (0.72-1.09)	1.05 (0.78-1.41)	0.292

[α]Hazard ratios (HRs) and 95% confidence intervals (95%CIs) were calculated by Cox proportional hazards models.

[β]Ranges for the quartiles of total dairy intake were 0-40.8 g/d, 44.9-109.8 g/d, 110.8-210.0 g/d, and 211.0-325.0 g/d in men.

[γ]*P*-trend was calculated using the median value of each category of total dairy intake and calculated by treating exposure as a continuous variable for subgroup dairy products freqency.

[δ]Adjusted for age (continuous),education level (junior high school or lower, high school, college or higher, or missing), BMI (<18.5kg/m^2, 18.5-24.9 kg/m^2, ≥25.0kg/m^2, or missing), smoking status (never, former, <20 cigarettes/d, ≥20 cigarettes/d, or missing), alcohol drinking status (current, never, former, or missing), history of hypertension (yes, or no), history of diabetes (yes, or no), energy intake (sex-specific tertiles or missing), protein intake (sex-specific tertiles or missing), fish intake (sex-specific tertiles or missing), and vegetable and fruit intake (sex-specific tertiles or missing).

e-Table 4. Association between total dairy intake and mortality after excluding death ascertained in the initial three years among Women (n=17,513)[α]

	Quartile of total dairy intake				*P*-trend [β]
	Q1	Q2	Q3	Q4	
All-cause mortality [γ]	1.00 (ref.)	0.97 (0.86-1.09)	1.04 (0.93-1.16)	0.98 (0.87-1.10)	0.752
Cancer mortality [γ]	1.00 (ref.)	0.85 (0.69-1.05)	0.97 (0.80-1.17)	1.00 (0.82-1.22)	0.896
CVD mortality [γ]	1.00 (ref.)	1.01 (0.80-1.27)	1.03 (0.83-1.27)	0.92 (0.73-1.16)	0.769
	Milk intake frequency				
	Almost never/ 1-2 times/mo	1-2 times/wk	3-4 times/wk	Almost daily	
All-cause mortality [γ]	1.00 (*ref.*)	1.01 (0.87-1.16)	0.95 (0.83-1.09)	1.01 (0.91-1.13)	0.831
Cancer mortality [γ]	1.00 (*ref.*)	0.94 (0.73-1.21)	0.84 (0.65-1.07)	0.98 (0.82-1.18)	0.955
CVD mortality [γ]	1.00 (*ref.*)	1.13 (0.85-1.50)	1.04 (0.79-1.37)	1.02 (0.83-1.26)	0.957
	Yogurt intake frequency				
	Almost never	1-2 times/mo	1-2 times/wk	3 times/wk/ Almost daily	
All-cause mortality [γ]	1.00 (*ref.*)	0.91 (0.82-1.01)	0.93 (0.84-1.04)	0.91 (0.80-1.03)	0.105
Cancer mortality [γ]	1.00 (*ref.*)	0.96 (0.80-1.15)	0.96 (0.79-1.16)	1.09 (0.88-1.33)	0.633
CVD mortality [γ]	1.00 (*ref.*)	0.90 (0.74-1.11)	0.90 (0.72-1.11)	0.86 (0.67-1.10)	0.185
	Cheese intake frequency				
	Almost never	1-2 times/mo	1-2 times/wk	3 times/wk/ Almost daily	
All-cause mortality [γ]	1.00 (*ref.*)	0.89 (0.81-0.97)	0.88 (0.78-1.00)	0.87 (0.72-1.05)	0.011
Cancer mortality [γ]	1.00 (*ref.*)	0.95 (0.80-1.12)	1.04 (0.84-1.28)	0.92 (0.67-1.28)	0.833
CVD mortality [γ]	1.00 (*ref.*)	0.89 (0.73-1.07)	0.94 (0.74-1.21)	0.98 (0.68-1.39)	0.556

[α]Hazard ratios (HRs) and 95% confidence intervals (95%CIs) were calculated by Cox proportional hazards models.

[β]Ranges for the quartiles of total dairy intake were 0-49.4 g/d, 49.9-205.0 g/d, 210.0-224.5 g/d, and 229.3-310.0 g/d in women.

[γ]*P*-trend was calculated using the median value of each category of total dairy intake and calculated by treating exposure as a continuous variable for subgroup dairy products freqency.

[δ]Adjusted for age (continuous),education level (junior high school or lower, high school, college or higher, or missing), BMI (<18.5kg/m^2, 18.5-24.9 kg/m^2, ≥25.0kg/m^2, or missing), smoking status (never, former, <20 cigarettes/d, ≥20 cigarettes/d, or missing), alcohol drinking status (current, never, former, or missing), history of hypertension (yes, or no), history of diabetes (yes, or no), energy intake (sex-specific tertiles or missing), protein intake (sex-specific tertiles or missing), fish intake (sex-specific tertiles or missing), and vegetable and fruit intake (sex-specific tertiles or missing).

e-Table 5. Association between total dairy intake and mortality in stratified analysis (n=34,161).

		Quartile of total dairy intake				P-trend[β]	P-interaction[γ]
		Q1	Q2	Q3	Q4		
**Men**							
All-cause mortality							
Age	<50 y (n=8,116)	1.00 (*ref.*)	0.91 (0.77-1.08)	0.91 (0.75-1.09)	0.93 (0.79-1.11)	0.470	0.778
	≥50 y (n=8,449)	1.00 (*ref.*)	0.94 (0.85-1.04)	1.02 (0.92-1.12)	0.92 (0.84-1.01)	0.618	
BMI	<25 kg/m^2 (n=11,598)	1.00 (*ref.*)	0.95 (0.86-1.05)	1.05 (0.94-1.16)	0.97 (0.88-1.07)	0.587	0.035
	≥25 kg/m^2 (n=4,483)	1.00 (*ref.*)	0.93 (0.79-1.10)	0.87 (0.73-1.04)	0.83 (0.70-0.98)	0.024	
Currently smoking	No (n=6,310)	1.00 (*ref.*)	1.05 (0.90-1.23)	0.99 (0.84-1.17)	0.90 (0.77-1.05)	0.119	0.402
	Yes (n=9,989)	1.00 (*ref.*)	0.91 (0.82-1.01)	0.98 (0.88-1.09)	0.94 (0.85-1.04)	0.819	
Cancer mortality							
Age	<50 y (n=8,116)	1.00 (*ref.*)	0.88 (0.68-1.15)	0.92 (0.69-1.24)	0.87 (0.67-1.15)	0.527	0.763
	≥50 y (n=8,449)	1.00 (*ref.*)	1.02 (0.87-1.19)	0.99 (0.84-1.16)	0.94 (0.80-1.09)	0.270	
BMI	<25 kg/m^2 (n=11,598)	1.00 (*ref.*)	1.02 (0.87-1.20)	1.02 (0.87-1.21)	0.98 (0.84-1.15)	0.405	0.131
	≥25 kg/m^2 (n=4,483)	1.00 (*ref.*)	0.93 (0.71-1.21)	0.83 (0.63-1.11)	0.75 (0.57-0.99)	0.039	
Currently smoking	No (n=6,310)	1.00 (*ref.*)	1.18 (0.91-1.54)	1.05 (0.80-1.38)	0.94 (0.73-1.21)	0.338	0.944
	Yes (n=9,989)	1.00 (*ref.*)	0.93 (0.79-1.09)	0.93 (0.79-1.10)	0.92 (0.78-1.08)	0.401	
CVD mortality							
Age	<50 y (n=8,116)	1.00 (*ref.*)	0.76 (0.53-1.08)	0.71 (0.47-1.06)	0.91 (0.65-1.29)	0.549	0.538
	≥50 y (n=8,449)	1.00 (*ref.*)	0.91 (0.74-1.12)	1.09 (0.89-1.32)	0.94 (0.78-1.15)	0.656	
BMI	<25 kg/m^2 (n=11,598)	1.00 (*ref.*)	0.92 (0.74-1.14)	1.09 (0.88-1.36)	0.96 (0.78-1.18)	0.551	0.631
	≥25 kg/m^2 (n=4,483)	1.00 (*ref.*)	0.81 (0.58-1.13)	0.82 (0.58-1.15)	0.94 (0.69-1.29)	0.766	
Currently smoking	No (n=6,310)	1.00 (*ref.*)	0.97 (0.70-1.34)	0.86 (0.62-1.20)	0.88 (0.65-1.19)	0.287	0.570
	Yes (n=9,989)	1.00 (*ref.*)	0.84 (0.67-1.03)	1.03(0.83-1.27)	0.96 (0.78-1.18)	0.520	
**Women**							
All-cause mortality							
Age	<50 y (n=7,942)	1.00 (*ref.*)	1.22 (0.96-1.54)	1.09 (0.84-1.41)	1.13 (0.88-1.46)	0.536	0.789
	≥50 y (n=9,654)	1.00 (*ref.*)	0.87 (0.76-1.00)	1.08 (0.96-1.21)	0.97 (0.85-1.10)	0.376	
BMI	<25 kg/m^2 (n=12,000)	1.00 (*ref.*)	0.96 (0.83-1.12)	1.06 (0.93-1.22)	1.01 (0.87-1.16)	0.474	0.551
	≥25 kg/m^2 (n=5,122)	1.00 (*ref.*)	0.98 (0.81-1.19)	0.99 (0.83-1.19)	0.95 (0.78-1.16)	0.736	
Currently smoking	No (n=13,629)	1.00 (*ref.*)	0.93 (0.81-1.07)	0.98 (0.87-1.12)	0.93 (0.81-1.06)	0.510	0.095
	Yes (n=1,195)	1.00 (*ref.*)	1.39 (0.95-2.05)	1.35 (0.92-1.98)	1.33 (0.88-2.00)	0.116	
Cancer mortality							
Age	<50 y (n=7,942)	1.00 (*ref.*)	1.23 (0.85-1.78)	0.87 (0.57-1.33)	0.91 (0.60-1.38)	0.352	0.128
	≥50 y (n=9,654)	1.00 (*ref.*)	0.68 (0.53-0.88)	1.02 (0.83-1.25)	1.02 (0.82-1.28)	0.341	
BMI	<25 kg/m^2 (n=12,000)	1.00 (*ref.*)	0.82 (0.63-1.07)	1.13 (0.89-1.43)	1.08 (0.85-1.38)	0.163	0.061
	≥25 kg/m^2 (n=5,122)	1.00 (*ref.*)	0.88 (0.63-1.23)	0.71 (0.51-0.99)	0.86 (0.61-1.20)	0.082	
Currently smoking	No (n=13,629)	1.00 (*ref.*)	0.84 (0.66-1.06)	0.90 (0.72-1.12)	0.99 (0.79-1.24)	0.748	0.562
	Yes (n=1,195)	1.00 (*ref.*)	1.02 (0.48-2.15)	1.29 (0.65-2.56)	1.23 (0.58-2.59)	0.433	

CVD mortality							
Age	<50 y (n=7,942)	1.00 (*ref.*)	1.12 (0.63-2.00)	1.13 (0.62-2.06)	1.59 (0.91-2.77)	0.169	0.090
	≥50 y (n=9,654)	1.00 (*ref.*)	0.97 (0.75-1.25)	1.10 (0.88-1.38)	0.85 (0.66-1.10)	0.852	
BMI	<25 kg/m^2 (n=12,000)	1.00 (*ref.*)	1.14 (0.84-1.54)	1.09 (0.82-1.44)	1.02 (0.75-1.38)	0.834	0.648
	≥25 kg/m^2 (n=5,122)	1.00 (*ref.*)	0.86 (0.59-1.25)	0.99 (0.71-1.38)	0.85 (0.58-1.24)	0.689	
Currently smoking	No (n=13,629)	1.00 (*ref.*)	0.96 (0.73-1.27)	1.01 (0.79-1.31)	0.90 (0.68-1.19)	0.742	0.172
	Yes (n=1,195)	1.00 (*ref.*)	1.86 (0.91-3.82)	1.87 (0.92-3.78)	1.19 (0.53-2.69)	0.293	

[α]Hazard ratios (HRs) and 95% confidence intervals (95%CIs) were calculated by Cox proportional hazards models.

[β] *P*-trend was calculated as per 100g/d increment of total dairy intake.

[γ]Adjusted for age (continuous), education level (junior high school or lower, high school, college or higher, or missing), BMI (<18.5kg/m^2, 18.5-24.9 kg/m^2, ≥25.0kg/m^2, or missing), smoking status (never, former, <20 cigarettes/d, ≥20 cigarettes/d, or missing), alcohol drinking status (current, never, former, or missing), history of hypertension (yes, or no), history of diabetes (yes, or no), energy intake (sex-specific tertiles or missing), fish intake (sex-specific tertiles or missing), and vegetable and fruit intake (sex-specific tertiles or missing) except the stratified variable.

e-Table 6. Association between milk intake and mortality in stratified analysis (n=34,161).

		Milk intake frequency					
		Almost never/ 1-2 times/mo	1-2 times/wk	3-4 times/wk	Almost daily	*P*-trend[β]	*P*-interaction[γ]
**Men**							
All-cause mortality							
Age	< y (n=8,116)	1.00 (*ref.*)	0.85 (0.70-1.03)	0.91 (0.76-1.10)	0.95 (0.81-1.11)	0.768	0.891
	≥ y (n=8,449)	1.00 (*ref.*)	1.00 (0.89-1.12)	0.91 (0.81-1.02)	0.98 (0.90-1.07)	0.547	
BMI	<25 kg/m^2 (n=11,598	1.00 (*ref.*)	0.95 (0.85-1.07)	0.97 (0.86-1.09)	1.01 (0.92-1.10)	0.704	0.081
	≥25 kg/m^2 (n=4,483)	1.00 (*ref.*)	0.99 (0.82-1.19)	0.84 (0.69-1.02)	0.88 (0.76-1.03)	0.061	
Currently smoking	No (n=6,310)	1.00 (*ref.*)	1.18 (0.98-1.40)	0.93 (0.78-1.12)	0.93 (0.80-1.07)	0.059	0.197
	Yes (n=9,989)	1.00 (*ref.*)	0.88 (0.78-0.99)	0.92 (0.82-1.04)	0.98 (0.90-1.08)	0.969	
Cancer mortality							
Age	< y (n=8,116)	1.00 (*ref.*)	0.78 (0.57-1.06)	0.94 (0.7-1.27)	0.91 (0.71-1.16)	0.677	0.941
	≥ y (n=8,449)	1.00 (*ref.*)	1.05 (0.88-1.25)	1.02 (0.86-1.22)	0.95 (0.82-1.09)	0.327	
BMI	<25 kg/m^2 (n=11,598	1.00 (*ref.*)	1.02 (0.85-1.23)	1.02 (0.85-1.23)	0.99 (0.86-1.14)	0.797	0.181
	≥25 kg/m^2 (n=4,483)	1.00 (*ref.*)	0.91 (0.67-1.23)	1.01 (0.75-1.35)	0.78 (0.61-1.00)	0.060	
Currently smoking	No (n=6,310)	1.00 (*ref.*)	1.35 (1.01-1.80)	1.21 (0.91-1.61)	0.93 (0.74-1.18)	0.175	0.611
	Yes (n=9,989)	1.00 (*ref.*)	0.86 (0.71-1.03)	0.95 (0.79-1.14)	0.93 (0.81-1.07)	0.507	
CVD mortality							
Age	< y (n=8,116)	1.00 (*ref.*)	0.61 (0.40-0.94)	0.84 (0.57-1.24)	0.90 (0.65-1.24)	0.874	0.810
	≥ y (n=8,449)	1.00 (*ref.*)	0.99 (0.79-1.25)	0.93 (0.73-1.17)	1.05 (0.88-1.25)	0.584	
BMI	<25 kg/m^2 (n=11,598	1.00 (*ref.*)	0.89 (0.69-1.15)	1.08 (0.85-1.38)	1.04 (0.86-1.26)	0.450	0.689
	≥25 kg/m^2 (n=4,483)	1.00 (*ref.*)	0.86 (0.59-1.26)	0.65 (0.44-0.98)	0.97 (0.73-1.30)	0.916	
Currently smoking	No (n=6,310)	1.00 (*ref.*)	1.03 (0.71-1.48)	0.87 (0.60-1.26)	0.88 (0.67-1.17)	0.302	0.467
	Yes (n=9,989)	1.00 (*ref.*)	0.81 (0.63-1.04)	0.93 (0.73-1.19)	1.05 (0.87-1.26)	0.368	
**Women**							
All-cause mortality							
Age	<50 y (n=7,942)	1.00 (*ref.*)	0.97 (0.72-1.31)	1.24 (0.94-1.64)	1.07 (0.84-1.36)	0.451	0.669
	≥50 y (n=9,654)	1.00 (*ref.*)	0.97 (0.82-1.14)	0.84 (0.72-0.98)	1.03 (0.91-1.15)	0.545	
BMI	<25 kg/m^2 (n=12,000	1.00 (*ref.*)	0.87 (0.72-1.05)	0.90 (0.75-1.07)	0.99 (0.87-1.13)	0.774	0.975
	≥25 kg/m^2 (n=5,122)	1.00 (*ref.*)	1.25 (0.99-1.58)	1.05 (0.84-1.32)	1.06 (0.88-1.26)	0.948	
Currently smoking	No (n=13,629)	1.00 (*ref.*)	1.00 (0.84-1.18)	0.92 (0.78-1.08)	0.96 (0.85-1.09)	0.477	0.067
	Yes (n=1,195)	1.00 (*ref.*)	0.95 (0.59-1.53)	1.61 (1.02-2.53)	1.29 (0.91-1.83)	0.082	
Cancer mortality							
Age	<50 y (n=7,942)	1.00 (*ref.*)	1.14 (0.73-1.78)	1.11 (0.71-1.73)	0.86 (0.58-1.26)	0.291	0.127
	≥50 y (n=9,654)	1.00 (*ref.*)	0.79 (0.58-1.07)	0.71 (0.53-0.94)	1.02 (0.83-1.25)	0.478	
BMI	<25 kg/m^2 (n=12,000	1.00 (*ref.*)	0.72 (0.51-1.00)	0.80 (0.59-1.08)	1.04 (0.83-1.30)	0.289	0.107
	≥25 kg/m^2 (n=5,122)	1.00 (*ref.*)	1.30 (0.88-1.90)	0.87 (0.59-1.31)	0.84 (0.62-1.15)	0.098	
Currently smoking	No (n=13,629)	1.00 (*ref.*)	0.97 (0.73-1.28)	0.85 (0.64-1.11)	0.96 (0.78-1.18)	0.684	0.530
	Yes (n=1,195)	1.00 (*ref.*)	1.34 (0.61-2.99)	0.90 (0.32-2.51)	1.34 (0.71-2.53)	0.443	

CVD mortality							
Age	<50 y (n=7,942)	1.00 (*ref.*)	0.58 (0.25-1.33)	1.45 (0.77-2.72)	1.32 (0.77-2.26)	0.108	0.078
	≥50 y (n=9,654)	1.00 (*ref.*)	1.19 (0.88-1.61)	0.94 (0.70-1.27)	1.03 (0.82-1.29)	0.948	
BMI	<25 kg/m^2 (n=12,000	1.00 (*ref.*)	1.12 (0.77-1.62)	1.14 (0.79-1.63)	1.06 (0.80-1.41)	0.774	0.777
	≥25 kg/m^2 (n=5,122)	1.00 (*ref.*)	1.12 (0.72-1.75)	0.96 (0.62-1.49)	1.01 (0.72-1.41)	0.918	
Currently smoking	No (n=13,629)	1.00 (*ref.*)	0.98 (0.69-1.38)	1.00 (0.72-1.37)	0.98 (0.76-1.25)	0.863	0.122
	Yes (n=1,195)	1.00 (*ref.*)	1.29 (0.52-3.16)	2.44 (1.06-5.60)	1.58 (0.80-3.11)	0.161	

[α]Hazard ratios (HRs) and 95% confidence intervals (95%CIs) were calculated by Cox proportional hazards models.

[β] *P*-trend was calculated by treating exposure as a continuous variable.

[γ]Model 1 was adjusted for age (continuous),education level (junior high school or lower, high school, college or higher, or missing), BMI (<18.5kg/m^2, 18.5-24.9 kg/m^2, ≥25.0kg/m^2, or missing), smoking status (never, former, <20 cigarettes/d, ≥20 cigarettes/d, or missing), alcohol drinking status (current, never, former, or missing), history of hypertension (yes, or no), history of diabetes (yes, or no), energy intake (sex-specific tertiles or missing), fish intake (sex-specific tertiles or missing), and vegetable and fruit intake (sex-specific tertiles or missing) except the stratified variable.

e-Table 7. Association between yogurt intake and mortality in stratified analysis (n=34,161).

		Yogurt intake frequency					
		Almost never	1-2 times/mo	1-2 times/wk	3 times/wk/ Almost daily	*P*-trend$^{\beta}$	*P*-interaction$^{\gamma}$
**Men**							
All-cause mortality							
Age	<50 y (n=8,116)	1.00 (*ref.*)	0.81 (0.69-0.95)	0.81 (0.66-0.99)	1.23 (0.97-1.56)	0.579	0.747
	≥50 y (n=8,449)	1.00 (*ref.*)	0.91 (0.84-1.00)	0.90 (0.80-1.01)	0.98 (0.86-1.12)	0.129	
BMI	<25 kg/m^2 (n=11,598	1.00 (*ref.*)	0.91 (0.86-1.03)	0.94 (0.83-1.06)	1.06 (0.93-1.21)	0.912	0.146
	≥25 kg/m^2 (n=4,483)	1.00 (*ref.*)	0.81 (0.70-0.95)	0.82 (0.68-1.00)	0.93 (0.72-1.20)	0.041	
Currently smoking	No (n=6,310)	1.00 (*ref.*)	0.98 (0.86-1.11)	0.96 (0.82-1.14)	1.15 (0.96-1.38)	0.414	0.020
	Yes (n=9,989)	1.00 (*ref.*)	0.88 (0.80-0.97)	0.86 (0.75-0.98)	0.95 (0.81-1.10)	0.022	
Cancer mortality							
Age	<50 y (n=8,116)	1.00 (*ref.*)	0.68 (0.52-0.88)	0.88 (0.65-1.21)	1.15 (0.78-1.71)	0.551	0.650
	≥50 y (n=8,449)	1.00 (*ref.*)	0.96 (0.84-1.10)	0.97 (0.81-1.17)	0.99 (0.80-1.23)	0.759	
BMI	<25 kg/m^2 (n=11,598	1.00 (*ref.*)	0.92 (0.80-1.06)	1.06 (0.88-1.27)	0.98 (0.79-1.22)	0.966	0.715
	≥25 kg/m^2 (n=4,483)	1.00 (*ref.*)	0.86 (0.67-1.10)	0.81 (0.58-1.12)	1.12 (0.76-1.65)	0.549	
Currently smoking	No (n=6,310)	1.00 (*ref.*)	1.04 (0.84-1.29)	0.98 (0.75-1.28)	1.09 (0.79-1.48)	0.740	0.238
	Yes (n=9,989)	1.00 (*ref.*)	0.85 (0.73-0.99)	0.96 (0.79-1.17)	0.96 (0.75-1.22)	0.351	
CVD mortality							
Age	<50 y (n=8,116)	1.00 (*ref.*)	0.80 (0.58-1.11)	0.68 (0.43-1.08)	1.11 (0.67-1.86)	0.349	0.636
	≥50 y (n=8,449)	1.00 (*ref.*)	0.94 (0.79-1.13)	0.95 (0.77-1.21)	0.95 (0.72-1.25)	0.583	
BMI	<25 kg/m^2 (n=11,598	1.00 (*ref.*)	0.97 (0.80-1.17)	0.89 (0.69-1.16)	0.97 (0.73-1.30)	0.522	0.504
	≥25 kg/m^2 (n=4,483)	1.00 (*ref.*)	0.88 (0.66-1.19)	1.01 (0.71-1.44)	1.06 (0.66-1.70)	0.970	
Currently smoking	No (n=6,310)	1.00 (*ref.*)	0.98 (0.75-1.29)	1.09 (0.79-1.51)	1.23 (0.86-1.77)	0.283	0.015
	Yes (n=9,989)	1.00 (*ref.*)	0.93 (0.77-1.13)	0.83 (0.64-1.09)	0.80 (0.56-1.12)	0.073	
**Women**							
All-cause mortality							
Age	<50 y (n=7,942)	1.00 (*ref.*)	1.00 (0.80-1.26)	1.02 (0.80-1.29)	1.11 (0.85-1.45)	0.500	0.062
	≥50 y (n=9,654)	1.00 (*ref.*)	0.87 (0.77-0.97)	0.90 (0.80-1.01)	0.84 (0.73-0.96)	0.009	
BMI	<25 kg/m^2 (n=12,000	1.00 (*ref.*)	0.92 (0.81-1.05)	0.95 (0.83-1.09)	0.92 (0.79-1.07)	0.288	0.731
	≥25 kg/m^2 (n=5,122)	1.00 (*ref.*)	0.94 (0.79-1.12)	0.96 (0.79-1.16)	0.94 (0.76-1.15)	0.517	
Currently smoking	No (n=13,629)	1.00 (*ref.*)	0.92 (0.81-1.04)	0.91 (0.81-1.04)	0.92 (0.79-1.06)	0.143	0.479
	Yes (n=1,195)	1.00 (*ref.*)	0.89 (0.62-1.29)	1.16 (0.79-1.71)	0.96 (0.61-1.50)	0.821	
Cancer mortality							
Age	<50 y (n=7,942)	1.00 (*ref.*)	1.23 (0.85-1.79)	0.99 (0.66-1.48)	1.42 (0.92-2.20)	0.304	0.534
	≥50 y (n=9,654)	1.00 (*ref.*)	0.89 (0.72-1.09)	0.97 (0.78-1.20)	1.00 (0.79-1.26)	0.971	
BMI	<25 kg/m^2 (n=12,000	1.00 (*ref.*)	0.97 (0.78-1.22)	0.93 (0.73-1.18)	1.08 (0.84-1.39)	0.790	0.349
	≥25 kg/m^2 (n=5,122)	1.00 (*ref.*)	1.12 (0.82-1.52)	1.12 (0.80-1.56)	1.21 (0.85-1.72)	0.295	
Currently smoking	No (n=13,629)	1.00 (*ref.*)	0.97 (0.78-1.19)	1.02 (0.82-1.27)	1.12 (0.89-1.42)	0.366	0.770
	Yes (n=1,195)	1.00 (*ref.*)	1.19 (0.62-2.28)	0.97 (0.44-2.13)	1.30 (0.57-2.95)	0.659	

CVD mortality							
Age	<50 y (n=7,942)	1.00 (*ref.*)	0.56 (0.33-0.97)	0.90 (0.54-1.48)	0.92 (0.52-1.62)	0.978	0.278
	≥50 y (n=9,654)	1.00 (*ref.*)	0.98 (0.79-1.21)	0.86 (0.68-1.09)	0.80 (0.62-1.05)	0.069	
BMI	<25 kg/m^2 (n=12,000	1.00 (*ref.*)	0.94 (0.72-1.22)	0.97 (0.74-1.28)	0.94 (0.69-1.27)	0.697	0.402
	≥25 kg/m^2 (n=5,122)	1.00 (*ref.*)	0.87 (0.63-1.20)	0.83 (0.58-1.19)	0.77 (0.51-1.15)	0.152	
Currently smoking	No (n=13,629)	1.00 (*ref.*)	0.91 (0.71-1.16)	0.90 (0.70-1.16)	0.88 (0.66-1.17)	0.302	0.912
	Yes (n=1,195)	1.00 (*ref.*)	1.18 (0.63-2.21)	1.21 (0.60-2.44)	0.37 (0.13-1.01)	0.172	

[α]Hazard ratios (HRs) and 95% confidence intervals (95%CIs) were calculated by Cox proportional hazards models.

[β] *P*-trend was calculated by treating exposure as a continuous variable.

[γ]Model 1 was adjusted for age (continuous),education level (junior high school or lower, high school, college or higher, or missing), BMI (<18.5kg/m^2, 18.5-24.9 kg/m^2, ≥25.0kg/m^2, or missing), smoking status (never, former, <20 cigarettes/d, ≥20 cigarettes/d, or missing), alcohol drinking status (current, never, former, or missing), history of hypertension (yes, or no), history of diabetes (yes, or no), energy intake (sex-specific tertiles or missing), fish intake (sex-specific tertiles or missing), and vegetable and fruit intake (sex-specific tertiles or missing) except the stratified variable.

e-Table 8. Association between cheese intake and mortality in stratified analysis (n=34,161).

		Cheese intake frequency					
		Almost never	1-2 times/mo	1-2 times/wk	3 times/wk/ Almost daily	*P*-trend[β]	*P*-interaction[γ]
**Men**							
All-cause mortality							
Age	<50 y (n=8,116)	1.00 (*ref.*)	0.93 (0.81-1.06)	0.96 (0.79-1.16)	0.99 (0.72-1.36)	0.572	0.939
	≥50 y (n=8,449)	1.00 (*ref.*)	0.87 (0.81-0.95)	0.94 (0.84-1.05)	1.08 (0.92-1.27)	0.361	
BMI	<25 kg/m^2 (n=11,598	1.00 (*ref.*)	0.88 (0.81-0.96)	0.97 (0.86-1.08)	1.07 (0.91-1.27)	0.553	0.973
	≥25 kg/m^2 (n=4,483)	1.00 (*ref.*)	0.93 (0.82-1.06)	0.92 (0.75-1.13)	1.03 (0.76-1.41)	0.511	
Currently smoking	No (n=6,310)	1.00 (*ref.*)	0.93 (0.82-1.05)	0.96 (0.81-1.14)	1.02 (0.79-1.31)	0.643	0.973
	Yes (n=9,989)	1.00 (*ref.*)	0.88 (0.81-0.96)	0.96 (0.85-1.08)	1.04 (0.87-1.25)	0.364	
Cancer mortality							
Age	<50 y (n=8,116)	1.00 (*ref.*)	1.03 (0.83-1.27)	1.00 (0.73-1.37)	0.78 (0.43-1.41)	0.693	0.231
	≥50 y (n=8,449)	1.00 (*ref.*)	1.00 (0.88-1.13)	1.17 (0.99-1.39)	1.16 (0.90-1.50)	0.080	
BMI	<25 kg/m^2 (n=11,598	1.00 (*ref.*)	1.01 (0.89-1.15)	1.24 (1.05-1.47)	1.17 (0.90-1.52)	0.027	0.259
	≥25 kg/m^2 (n=4,483)	1.00 (*ref.*)	1.02 (0.83-1.26)	0.84 (0.60-1.19)	1.08 (0.64-1.79)	0.720	
Currently smoking	No (n=6,310)	1.00 (*ref.*)	1.13 (0.93-1.38)	1.14 (0.87-1.50)	1.09 (0.711.65)	0.290	0.655
	Yes (n=9,989)	1.00 (*ref.*)	0.96 (0.84-1.09)	1.15 (0.96-1.38)	1.06 (0.79-1.41)	0.338	
CVD mortality							
Age	<50 y (n=8,116)	1.00 (*ref.*)	0.94 (0.71-1.24)	0.86 (0.56-1.30)	0.93 (0.47-1.85)	0.498	0.888
	≥50 y (n=8,449)	1.00 (*ref.*)	0.79 (0.67-0.93)	0.85 (0.67-1.07)	1.04 (0.75-1.43)	0.155	
BMI	<25 kg/m^2 (n=11,598	1.00 (*ref.*)	0.77 (0.65-0.91)	0.86 (0.67-1.09)	0.93 (0.65-1.33)	0.081	0.285
	≥25 kg/m^2 (n=4,483)	1.00 (*ref.*)	1.00 (0.781.28)	0.84 (0.55-1.27)	1.21 (0.69-2.10)	0.924	
Currently smoking	No (n=6,310)	1.00 (*ref.*)	0.87 (0.68-1.11)	1.04 (0.75-1.44)	0.83 (0.48-1.44)	0.588	0.559
	Yes (n=9,989)	1.00 (*ref.*)	0.82 (0.69-0.98)	0.76 (0.59-1.00)	1.03 (0.72-1.46)	0.093	
**Women**							
All-cause mortality							
Age	<50 y (n=7,942)	1.00 (*ref.*)	1.03 (0.84-1.25)	1.00 (0.78-1.29)	0.95 (0.63-1.45)	0.928	0.160
	≥50 y (n=9,654)	1.00 (*ref.*)	0.84 (0.75-0.93)	0.81 (0.70-0.93)	0.87 (0.71-1.07)	0.001	
BMI	<25 kg/m^2 (n=12,000	1.00 (*ref.*)	0.94 (0.83-1.05)	0.89 (0.76-1.04)	0.88 (0.70-1.10)	0.077	0.839
	≥25 kg/m^2 (n=5,122)	1.00 (*ref.*)	0.83 (0.71-0.98)	0.84 (0.67-1.06)	0.92 (0.66-1.29)	0.089	
Currently smoking	No (n=13,629)	1.00 (*ref.*)	0.89 (0.80-1.00)	0.83 (0.71-0.96)	0.91 (0.73-1.13)	0.016	0.401
	Yes (n=1,195)	1.00 (*ref.*)	1.06 (0.77-1.47)	0.93 (0.60-1.45)	0.95 (0.50-1.78)	0.807	
Cancer mortality							
Age	<50 y (n=7,942)	1.00 (*ref.*)	1.22 (0.89-1.68)	1.24 (0.82-1.85)	0.96 (0.46-1.85)	0.440	0.353
	≥50 y (n=9,654)	1.00 (*ref.*)	0.86 (0.71-1.04)	0.94 (0.74-1.20)	0.95 (0.65-1.34)	0.441	
BMI	<25 kg/m^2 (n=12,000	1.00 (*ref.*)	0.95 (0.77-1.16)	0.99 (0.77-1.28)	1.03 (0.71-1.49)	0.999	0.998
	≥25 kg/m^2 (n=5,122)	1.00 (*ref.*)	0.99 (0.75-1.32)	1.12 (0.77-1.62)	0.69 (0.35-1.37)	0.756	
Currently smoking	No (n=13,629)	1.00 (*ref.*)	0.92 (0.76-1.11)	1.02 (0.80-1.29)	1.07 (0.75-1.52)	0.864	0.480
	Yes (n=1,195)	1.00 (*ref.*)	1.16 (0.64-2.11)	0.95 (0.40-2.29)	0.60 (0.14-1.14)	0.684	

CVD mortality							
Age	<50 y (n=7,942)	1.00 (*ref.*)	0.99 (0.63-1.56)	1.31 (0.78-2.22)	1.50 (0.69-3.27)	0.224	0.041
	≥50 y (n=9,654)	1.00 (*ref.*)	0.88 (0.71-1.08)	0.80 (0.60-1.05)	0.90 (0.61-1.33)	0.123	
BMI	<25 kg/m^2 (n=12,000	1.00 (*ref.*)	1.05 (0.82-1.34)	1.07 (0.79-1.45)	0.99 (0.63-1.56)	0.788	0.303
	≥25 kg/m^2 (n=5,122)	1.00 (*ref.*)	0.72 (0.53-0.99)	0.71 (0.45-1.12)	1.00 (0.56-1.80)	0.178	
Currently smoking	No (n=13,629)	1.00 (*ref.*)	0.90 (0.72-1.13)	0.99 (0.74-1.32)	0.98 (0.64-1.52)	0.789	0.311
	Yes (n=1,195)	1.00 (*ref.*)	1.33 (0.73-2.41)	1.24 (0.58-2.65)	0.85 (0.28-2.65)	0.821	

[α]Hazard ratios (HRs) and 95% confidence intervals (95%CIs) were calculated by Cox proportional hazards models.

[β] *P*-trend was calculated by treating exposure as a continuous variable.

[γ]Model 1 was adjusted for age (continuous),education level (junior high school or lower, high school, college or higher, or missing), BMI (<18.5kg/m^2, 18.5-24.9 kg/m^2, ≥25.0kg/m^2, or missing), smoking status (never, former, <20 cigarettes/d, ≥20 cigarettes/d, or missing), alcohol drinking status (current, never, former, or missing), history of hypertension (yes, or no), history of diabetes (yes, or no), energy intake (sex-specific tertiles or missing), fish intake (sex-specific tertiles or missing), and vegetable and fruit intake (sex-specific tertiles or missing) except the stratified variable.

e-Table 9. Association between dairy intake and mortality after multiple imputation among men[α]

	Quartile of total dairy intake				*P*-trend [β]
	Q1	Q2	Q3	Q4	
All-cause mortality [γ]	1.00 (*ref.*)	0.96 (0.89-1.04)	0.98 (0.91-1.06)	0.95 (0.88-1.02)	0.214
Cancer mortality [γ]	1.00 (*ref.*)	0.99 (0.87-1.12)	0.99 (0.88-1.12)	0.95 (0.84-1.07)	0.421
CVD mortality [γ]	1.00 (*ref.*)	0.89 (0.77-1.03)	0.96 (0.83-1.11)	0.90 (0.76-1.06)	0.385
	Milk intake frequency				
	Almost never/ 1-2 times/mo	1-2 times/week	3-4 times/week	Almost daily	
All-cause mortality [γ]	1.00 (*ref.*)	0.97 (0.89-1.07)	0.95 (0.87-1.03)	0.97 (0.91-1.04)	0.462
Cancer mortality [γ]	1.00 (*ref.*)	0.98 (0.84-1.13)	1.01 (0.88-1.15)	0.97 (0.87-1.07)	0.569
CVD mortality [γ]	1.00 (*ref.*)	0.88 (0.75-1.04)	0.92 (0.77-1.10)	0.95 (0.83-1.09)	0.683
	Yogurt intake frequency				
	Almost never	1-2 times/mo	1-2 times/wk	3 times/wk/ Almost daily	
All-cause mortality [γ]	1.00 (*ref.*)	0.93 (0.87-1.00)	0.92 (0.83-1.02)	1.02 (0.91-1.14)	0.303
Cancer mortality [γ]	1.00 (*ref.*)	0.93 (0.83-1.05)	0.99 (0.83-1.18)	1.05 (0.90-1.23)	0.873
CVD mortality [γ]	1.00 (*ref.*)	0.93 (0.79-1.08)	0.90 (0.74-1.08)	0.98 (0.74-1.30)	0.430
	Cheese intake frequency				
	Almost never	1-2 times/mo	1-2 times/wk	3 times/wk/ Almost daily	
All-cause mortality [γ]	1.00 (*ref.*)	0.91 (0.85-0.98)	0.97 (0.87-1.08)	1.03 (0.91-1.17)	0.513
Cancer mortality [γ]	1.00 (*ref.*)	0.99 (0.88-1.11)	1.09 (0.93-1.27)	1.08 (0.86-1.37)	0.314
CVD mortality [γ]	1.00 (*ref.*)	0.89 (0.79-1.00)	0.90 (0.77-1.05)	1.02 (0.81-1.29)	0.269

[α]Pooled hazard ratios (HRs) and 95% confidence intervals (95%CIs) were calculated by Cox proportional hazards models using five imputed datasets.

[β]P-trend was calculated using the median value of each category of total dairy intake and calculated by treating exposure as a continuous variable for subgroup dairy products freqency.

[γ]Adjusted for age (continuous),education level (junior high school or lower, high school, college or higher, or missing), BMI (<18.5kg/m2, 18.5-24.9 kg/m2, ≥25.0kg/m2, or missing), smoking status (never, former, <20 cigarettes/d, ≥20 cigarettes/d, or missing), alcohol drinking status (current, never, former, or missing), history of hypertension (yes, or no), history of diabetes (yes, or no), energy intake (sex-specific tertiles or missing), fish intake (sex-specific tertiles or missing), and vegetable and fruit intake (sex-specific tertiles or missing).

e-Table 10. Association between dairy intake and mortality after multiple imputation among women [α]

	Quartile of total dairy intake				P-trend [β]
	Q1	Q2	Q3	Q4	
All-cause mortality [γ]	1.00 (ref.)	0.96 (0.87-1.07)	0.98 (0.90-1.08)	0.95 (0.86-1.04)	0.335
Cancer mortality [γ]	1.00 (ref.)	0.90 (0.74-1.08)	0.94 (0.79-1.13)	0.96 (0.82-1.14)	0.816
CVD mortality [γ]	1.00 (ref.)	1.00 (0.78-1.26)	0.99 (0.79-1.23)	0.88 (0.73-1.05)	0.173
	Milk intake frequency				
	Almost never/ 1-2 times/mo	1-2 times/wk	3-4 times/wk	Almost daily	
All-cause mortality [γ]	1.00 (*ref.*)	1.02 (0.88-1.18)	0.92 (0.82-1.04)	0.96 (0.88-1.06)	0.279
Cancer mortality [γ]	1.00 (*ref.*)	0.99 (0.80-1.24)	0.82 (0.67-1.01)	0.94 (0.81-1.11)	0.416
CVD mortality [γ]	1.00 (*ref.*)	1.10 (0.87-1.40)	0.99 (0.80-1.24)	0.96 (0.81-1.14)	0.381
	Yogurt intake frequency				
	Almost never	1-2 times/mo	1-2 times/wk	3 times/wk/ Almost daily	
All-cause mortality [γ]	1.00 (*ref.*)	0.93 (0.84-1.02)	0.94 (0.86-1.03)	0.94 (0.84-1.06)	0.226
Cancer mortality [γ]	1.00 (*ref.*)	0.97 (0.84-1.13)	0.98 (0.83-1.16)	1.07 (0.90-1.27)	0.546
CVD mortality [γ]	1.00 (*ref.*)	0.93 (0.76-1.13)	0.91 (0.76-1.09)	0.86 (0.67-1.10)	0.162
	Cheese intake frequency				
	Almost never	1-2 times/mo	1-2 times/wk	3 times/wk/ Almost daily	
All-cause mortality [γ]	1.00 (*ref.*)	0.91 (0.84-0.99)	0.91 (0.82-1.01)	0.93 (0.78-1.11)	0.029
Cancer mortality [γ]	1.00 (*ref.*)	0.96 (0.80-1.14)	1.02 (0.86-1.21)	0.94 (0.71-1.24)	0.780
CVD mortality [γ]	1.00 (*ref.*)	0.92 (0.79-1.08)	0.93 (0.72-1.20)	1.03 (0.75-1.43)	0.671

[α]Pooled hazard ratios (HRs) and 95% confidence intervals (95%CIs) were calculated by Cox proportional hazards models using five imputed datasets.

[β] P-trend was calculated using the median value of each category of total dairy intake and calculated by treating exposure as a continuous variable for subgroup dairy products freqency.

[γ]Adjusted for age (continuous),education level (junior high school or lower, high school, college or higher, or missing), BMI (<18.5kg/m^2, 18.5-24.9 kg/m^2, ≥25.0kg/m^2, or missing), smoking status (never, former, <20 cigarettes/d, ≥20 cigarettes/d, or missing), alcohol drinking status (current, never, former, or missing), history of hypertension (yes, or no), history of diabetes (yes, or no), energy intake (sex-specific tertiles or missing), fish intake (sex-specific tertiles or missing), and vegetable and fruit intake (sex-specific tertiles or missing).
